# Solvent Effects on Skin Penetration and Spatial Distribution of the Hydrophilic Nitroxide Spin Probe PCA Investigated by EPR

**DOI:** 10.1007/s12013-020-00908-3

**Published:** 2020-04-17

**Authors:** Pin Dong, Christian Teutloff, Jürgen Lademann, Alexa Patzelt, Monika Schäfer-Korting, Martina C. Meinke

**Affiliations:** 1 Department of Dermatology, Venereology and Allergology, Charité - Universitätsmedizin Berlin, corporate member of Freie Universität Berlin, Humboldt-Universität zu Berlin, and Berlin Institute of Health, Berlin, Germany; 2 Freie Universität Berlin, Institute of Pharmacy, Pharmacology and Toxicology, Berlin, Germany; 3 Freie Universität Berlin, Department of Physics, Institute of Experimental Physics, Berlin, Germany

**Keywords:** Corneocytes, Electron paramagnetic resonance, Small hydrophilic molecule, Skin lipids, Skin pathway

## Abstract

Oxidative stress occurs in extrinsic skin aging processes and diseases when the enhanced production of free radicals exceeds the homeostatic antioxidant capacity of the skin. The spin probe, 3-(carboxy)-2,2,5,5-tetramethylpyrrolidin-1-oxyl (PCA), is frequently used to study the cutaneous radical production by electron paramagnetic resonance (EPR) spectroscopy. This approach requires delivering PCA into the skin, yet solvent effects on the skin penetration and spatial distribution of PCA have not been thoroughly investigated. Three solvents of ethanol, phosphate-buffered saline (PBS) and ethanol-PBS (1:1) were studied. For both human and porcine skin ex vivo, the amount of PCA in the stratum corneum (SC) was the lowest when using ethanol and very similar for PBS and ethanol-PBS. The highest amount of PCA in the viable skin layers was detected for ethanol-PBS, yet it only took up less than 5% of the total amount. The majority of PCA was localized in the SC, among which PCA with high mobility was predominantly distributed in the hydrophilic microenvironment of corneocytes and PCA with lower mobility was mainly in the less hydrophilic microenvironment of intercellular skin lipids. A higher ethanol concentration in the solvent could improve the distribution of PCA in the hydrophilic microenvironments of the SC. The results suggest that ethanol-PBS (1:1) is best-suited for delivering most PCA deep into the skin. This work enhances the understanding of solvent effects on the skin penetration and distribution of PCA and supports the utilization of PCA in studying cutaneous radical production.

## Introduction

Oxidative stress plays a significant role in extrinsic skin aging processes [1] and diseases [2]. It occurs when the production of oxygen and nitrogen radicals [3] overwhelms the homeostatic antioxidant capacity of the skin [4]. Radicals are molecules with unpaired electrons, which can be detected by electron paramagnetic resonance (EPR) spectroscopy [5, 6]. However, a direct EPR detection of endogenous free radicals in the skin under physiological conditions remains challenging due to their short lifetime [7]. Therefore, alternative approaches are utilizing spin traps and probes to investigate the free radical production in the skin [8].

Spin traps scavenge reactive free radicals effectively and form more stable paramagnetic spin adducts to facilitate EPR measurements. Different radical species can be distinguished from their unique EPR spectra of spin adducts [9]. Spin traps are often influenced by impurity [10] and dissolving media [11]. They are also less sensitive and less reliable for the quantitative determination of radicals [12]. In comparison, spin probes are molecules with stable free radical character and can be reduced to EPR silent hydroxylamine by free radicals generated in the skin [13]. Therefore, the intensity reduction of spin probes correlates well with the production of free radicals. This approach is widely used to study skin oxidative stress [12–14]. The spin probe of 3-(carboxy)-2,2,5,5-tetramethylpyrrolidin-1-oxyl (PCA), with low toxicity and low irritation to the skin [15], is often used in medical and cosmetic studies [16–19], such as photodynamic therapy [20, 21], light-induced radical production and sunscreen development [13, 18, 19, 22, 23]. In these in vivo studies, the skin was incubated with PCA solution from the outermost SC, and in some applications, test products were applied subsequently. Then the radical formation under light irradiation was investigated by EPR. Therefore, the prerequisite of this approach is to deliver PCA into the skin.

In vivo application of PCA demands a formulation with simple and non-toxic compositions. Thus, PCA is often dissolved in phosphate-buffered saline (PBS), ethanol, or the mixture of PBS and ethanol [12, 18]. PBS is a non-toxic and well-suited solvent due to the hydrophilicity of PCA [24]. Ethanol is a widely used penetration enhancer [25]. The ethanol-PBS cosolvent system has been found superior to transport drugs into the skin compared to the unary solvents [26–29]. Yet, solvent effects on the skin penetration amount and depth of PCA, as well as the spatial distribution, have not been thoroughly investigated so far [13, 22]. In this work, the penetration of PCA into human and porcine skin delivered by the three solvents (ethanol, PBS, and ethanol-PBS 1:1) was quantified by EPR. Furthermore, the influences of these solvents on the spatial distribution of PCA in the skin were analyzed.

## Materials and Methods

PBS (Gibco^TM^) was purchased from ThermoFisher Scientific (Waltham, MA, USA). PCA (98%), ethanol (Uvasol^®^, for spectroscopy) and Triton X-100 (laboratory grade) were bought from Sigma-Aldrich (Merck, Darmstadt, Germany). Cyclohexane (Rotisolv^®^ HPLC) was bought from Carl Roth GmbH + Co. KG (Karlsruhe, Germany).

### Skin Samples

Excised human abdominal skin was donated by female volunteers with no medical history of dermatological diseases who underwent plastic surgery after informed written consent. The Ethics Committee of Charité-Universitätsmedizin Berlin approved the study in accordance with the principles expressed in the Declaration of Helsinki. After excision, the subcutaneous fatty tissue was removed from the skin specimen using a scalpel, and subsequently, the remaining skin samples were cleaned with PBS.

Porcine ears were obtained from a local slaughterhouse with the approval of the Commission of Consumer Protection and Agriculture, District Dahme-Spreewald, Germany. The ears were cleaned with cold tap water and gently dried with paper towels. The hairs were carefully cut with scissors without damaging the SC. All human and porcine skin samples were stored at 4 °C and used within 24 h.

### PCA Application and Incubation Protocol

For human skin, each skin sample was stretched and fixed with needles on a styrofoam plate and stripped with one tape to remove fatty substances on the skin surface. Six areas of each skin sample (*n* = 8) and each area in the size of 3 × 7 cm^2^ were prepared and examined by a magnifying glass to exclude samples with any damage, scars or stretch marks. Each area was placed on two stacked paper discs (Finn Chambers^®^, Φ 12 mm), leaving safety margins of about 9 mm to the skin border to avoid lateral penetration. Then 100 µl of 0.4% PCA solutions dissolved in ethanol, PBS, and ethanol-PBS 1:1 were pipetted onto the paper discs, respectively. Every solution was applied to two areas of each skin sample. After occlusion of the skin areas by Finn Chambers^®^ (Φ 12 mm), the skin samples were incubated for 40 min at 32 °C. This procedure was done in the same way as published in vivo studies [19, 23, 30]. To be noted, the concentration of PCA herein was reduced by half compared with the concentration used in these in vivo studies because of the limited solubility of PCA in PBS. For porcine ear skin, six areas of each pair (*n* = 6) were treated in the same procedure as for human skin.

### Skin Sample Preparation for EPR Measurements

After incubation, the paper discs were removed and subsequently all skin samples were subjected to 3 tape strippings because the high amount of PCA accumulated on the skin surface resulted in the spin-spin effect that depletes the EPR signal in EPR measurements [31]. Among the six areas of each skin sample, half of them exposed to the three PCA solutions, respectively, were removed the entire human stratum corneum (SC) by performing 4 times cyanoacrylate strippings (5 times for porcine skin) as previously described [32]. Afterward, all skin samples were dermatomed to a thickness of 300 µm (Aesculap, Tuttlingen, Germany) and punched into discs of 5 mm in diameter for EPR measurements. PCA in the non-stripped and cyanoacrylate-stripped skin samples represented the amount of PCA penetrated in the whole skin (i.e., the SC plus viable skin layers) and only in the viable skin layers, respectively. The difference between these two values was the amount of PCA in the SC.

### Incubation of PCA with Skin Lipids

To obtain the EPR spectrum of PCA in skin lipids, they were extracted from porcine ear skin by using a mixture of cyclohexane and ethanol (4:1, V/V) [33]. A 15 ml Falcon^TM^ centrifuge tube with an area of 2.27 cm^2^ was filled with 1 ml of the solvent mixture and then it was held firmly against the skin while being shaken for 1 min. About 30 skin areas were performed, and each area was extracted twice. The collected solvent was centrifuged at 10,000 rpm (Hettich AG, Switzerland) for 10 min to remove a few exfoliated corneocytes (precipitates). After overnight evaporating under a fume hood, the skin lipids were collected. Afterward, the skin lipids and PCA were dissolved in cyclohexane-ethanol (4:1) and evaporated again to obtain samples of PCA in skin lipids at a concentration of 0.001% (W/W).

### Incubation of PCA with Corneocytes

Corneocytes were prepared by the detergent scrub method [34, 35]. A fresh porcine ear placed in a glass Petri dish was rubbed by a polyester sponge soaked with 0.1% Triton X-100 in PBS. Any skin area was scrubbed 50 times and the washed fluid was sub-packed into 2 ml tubes, which were subsequently centrifuged at 10,000 rpm for 10 min. The supernatant was discarded, and the precipitated corneocytes in each tube were resuspended with 2 ml PBS. The procedure of centrifugation-resuspension was repeated 5 times to wash away Triton X-100 [36]. Afterward, the collected corneocytes were incubated with 200 µl of 0.4 % PCA in ethanol-PBS (1:1) for 40 min at 32 °C. After the incubation, PCA in the external medium was removed with PBS in the same way as removing Triton X-100, and the procedure was repeated ten times. The supernatant after each washing step and the precipitated corneocytes after 10 times washing were measured by EPR.

### EPR Measurements

All measurements were conducted with an X-band EPR spectrometer (Elexsys E500, Bruker BioSpin, Karlsruhe, Germany) at ambient temperature. An SHQE resonator (E4122011SHQE, Bruker Biospin, Germany) and a TMHS resonator (E2044500TMHS, Bruker BioSpin) were used, which were matched to a sample holder of a capillary (2.0/1.0 mm in o.d./i.d., Hirschmann Laborgeräte, Germany) and a tissue cell (ER 162TC-Q, Bruker Biospin,), respectively. The instrumental settings of microwave power (mW) and field modulation amplitude (mT) are summarized in Table 1. The field modulation frequency was 100 kHz in all measurements. Before measuring the samples, the quantification accuracy of the spectrometer was calibrated with the Alanine standard (Bruker, the standard spin number is 2.05 × 10^17^ ± 10%).

**Table 1:** EPR experiment settings

Samples	Sample holder	Resonator	EPR settings
Skin containing SC plus viable skin layers	Tissue cell	TMHS	0.1 mT, 0.6 mW
Skin containing only viable skin layers	Tissue cell	TMHS	0.3 mT, 6.3 mW
PCA in skin lipids	Tissue cell	TMHS	0.3 mT, 20 mW
PBS from the washing of corneocytes	Capillary	SHQE	0.3 mT, 20 mW
Corneocytes incubated with PCA	Capillary	SHQE	0.3 mT, 20 mW

The total numbers and concentrations of PCA were quantified by the SpinCount module in the Bruker Xepr software platform. EPR spectra of PCA in skin samples, skin lipids, and corneocytes were simulated using EasySpin [37], a toolbox package for Matlab (The MathWorks GmbH, Natick, MA, USA). The *chili* function [38] was used for the simulation, and the magnetic parameters of *g*-matrix and 14 N hyperfine coupling constant were referred to the published values [39].

### Statistical Analysis

Data are shown as mean ± standard error of the mean (SEM). Comparisons of the PCA transported into the human or porcine skin by the three solvents were evaluated by the nonparametric two-related samples Wilcoxon test. The differences of PCA penetrated into human and porcine skin using the same solution were determined through the nonparametric two-independent samples Mann–Whitney U test. The minimal significance level was set at *p* ≤ 0.05.

## Results and Discussion

### Skin Penetration of PCA

**Fig. 1:**
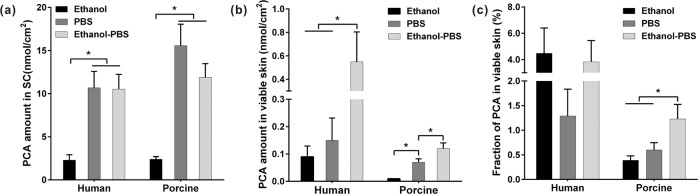
The amount of PCA in the (**a**) SC, (**b**) viable skin layers and (**c**) the fraction of PCA in the viable skin to the total amount of PCA in the skin after applied 0.4% PCA solution dissolved in ethanol, PBS and ethanol-PBS (1:1, V/V) to human skin (*n* = 8) and porcine ear skin (*n* = 6), respectively. The area of every skin biopsy was 0.20 cm^2^. The total amount of PCA in the skin is the sum of the amount of PCA in the SC and viable skin layers. Mean ± SEM, * indicates *p* < 0.05. No significant difference was found between human and porcine skin treated with the same PCA solution

The amounts of PCA transported into the SC and viable skin layers by ethanol, PBS and ethanol-PBS (1:1), respectively, are shown in Fig. 1a. For human skin, about 2.3 µg/cm^2^ PCA was found in the SC using ethanol, which is the lowest among the three solvents. Ethanol is a well-known penetration enhancer, whereas it showed little improvement for the skin penetration of PCA. This could be due to the fast evaporation of ethanol. Many white PCA precipitates were seen on the skin surface after the incubation, which were formed due to the evaporation of ethanol, even though an occlusive chamber was used to reduce the evaporation. The precipitation of PCA could hinder the cutaneous penetration of PCA. Thus, pure ethanol is not recommended to deliver PCA into the skin. In comparison, the amounts of PCA in the human SC were 4.5 times increased in both cases of PBS and ethanol-PBS (1:1). This is different from many published findings, which stated that the PBS-ethanol cosolvent was better than the pure solvents, e.g., ethanol or PBS [26–29]. PCA is a small hydrophilic molecule (186 g/mol) with a natural logarithmic partition coefficient of −1.8 [40]. With the hydration of the SC by PBS [41, 42], possibly the solubility of PCA in the SC was increased, and hence the penetration of PCA into the SC could be enhanced.

In addition, the amount of PCA in the human viable skin layers was also quantified. Figure 1b shows that the highest amount of PCA was delivered by ethanol-PBS (1:1). PBS facilitated slightly more PCA penetration into the viable skin compared to ethanol, even though there was no statistical significance in human skin due to high inter-donor variances. The results demonstrated the advantage of combining ethanol with PBS to transport more PCA into the viable skin layers. The addition of PBS not only reduced the ethanol evaporation [43] but also hydrated the SC; while ethanol could extract appreciable amounts of lipids from the SC or influenced the structures of both corneocytes and skin lipid lamellar, and consequently may lower the skin barrier function [44, 45]. It is noteworthy that the actual amount of PCA in the skin could be higher than the measured amount by EPR because the endogenous radicals originated from the cellular metabolism and some antioxidants in the skin such as ascorbic acid could consume some PCA [46, 47], although PCA hardly diffuses into the living cells due to its hydrophilicity [48].

For porcine skin, the amounts of PCA delivered into the SC by the three solvents were in the same order as human skin (ethanol-PBS ≈ PBS > ethanol). There was no difference in the amount of PCA in the SC between human and porcine skin subjected to the same PCA solution. The amounts of PCA in the porcine viable skin layers using the three solvents were also in a similar order as human skin, i.e., ethanol-PBS > PBS > ethanol. Although the absolute values were lower than those of human skin, there was no statistical difference between porcine and human skin treated with the same PCA solution. This indicates that porcine skin could be a good substitute for human skin to study the skin penetration of PCA.

The fractions of the amount of PCA in the viable skin layers to the total amount of PCA in the skin are shown in Fig. 1c. For human skin, the fractions were in the range of 1.3–4.9% when using the three solvents, and no statistical differences were found within the solvents due to the high inter-donor variation. For porcine skin, the highest fraction was about 1.2% in the case of ethanol-PBS, while it was less than 1% when using ethanol and PBS.

The above results show that PCA in the viable skin constituted less than 5% of the total penetration amount and more than 95% PCA accumulated in the SC. This is important information for studies of skin radical formation under light irradiation. First, PCA needs to be delivered to the depth of viable skin layers, because red light used in the photodynamic therapy and UVA to near-infrared light of the sun spectrum penetrate deep into the viable skin, meaning that free radicals would be induced in both the SC and viable skin layers [21, 49, 50]. If no PCA molecules penetrated into the viable skin layers, free radicals produced there could not react with PCA. Consequently, the measured amount of free radicals would be lower than the actual amount of free radicals.

This is particularly important for quantitative measurements of free radicals. The free radical threshold value in the human skin is about 3.5 × 10^12^ radicals/mg, beyond which all the endogenous antioxidants in the skin could be consumed [51]. External stimuli that induce oxidative stress in the skin, such as light irradiation, would generate free radicals above this threshold [12]. This means that more than 3.5 × 10^12^ of PCA molecules/mg should be delivered into the viable skin layers to ensure that most free radicals are reacted with PCA. By using ethanol, PBS and ethanol-PBS (1:1), the amount of PCA in the viable skin layers that normalized to the skin weight was about 1 × 10^12^, 5 × 10^12^ and 9 × 10^12^ radicals/mg, respectively, when 1 g/cm^3^ was roughly taken as the density of the skin [52]. Therefore, the amount of PCA in the viable skin delivered by ethanol-PBS (1:1) could be enough to determine the threshold.

Nevertheless, the amount of free radicals produced in the viable skin layers depends on the extent of applied external stimuli, such as the irradiation dose. A linear decay of PCA could be a good indication to assume that the amount of PCA transported into the skin is sufficient to detect all free radicals [23]. Otherwise, a few tape strippings can be used to slightly disturb the SC barrier before applying PCA solutions to human skin in vivo, through which the amount of PCA penetrating into the viable skin could be increased. Alternatively, longer incubation time, potent penetration enhancers, and increasing the concentration of PCA could be possible strategies to enhance the skin penetration of PCA. However, for in vivo studies, long incubation time would be poorly compliant, and potent penetration enhancers could have an issue of skin toxicity. In contrast, PBS as a safe solvent and ethanol as an FDA-approved solvent for skin application, are favored for in vivo studies. In addition, using a high concentration of PCA to increase the skin penetration of PCA could also increase the amount of PCA in the SC. Namely, the concentration of PCA in the SC is increased, which may cause the spin-spin effect that interferes EPR measurements [31]. But it should be mentioned that viable skin cells (e.g., keratinocytes and fibroblasts) and reconstructed human skin models are sensitive to ethanol. The concentration of ethanol above 3% could make half of the cells die [53]. Therefore, when PCA is used for skin cells or reconstructed models, only PBS should be used as the solvent.

### Distributions of PCA in Skin

#### Microenvironments of PCA in the whole skin layers

**Fig. 2:**
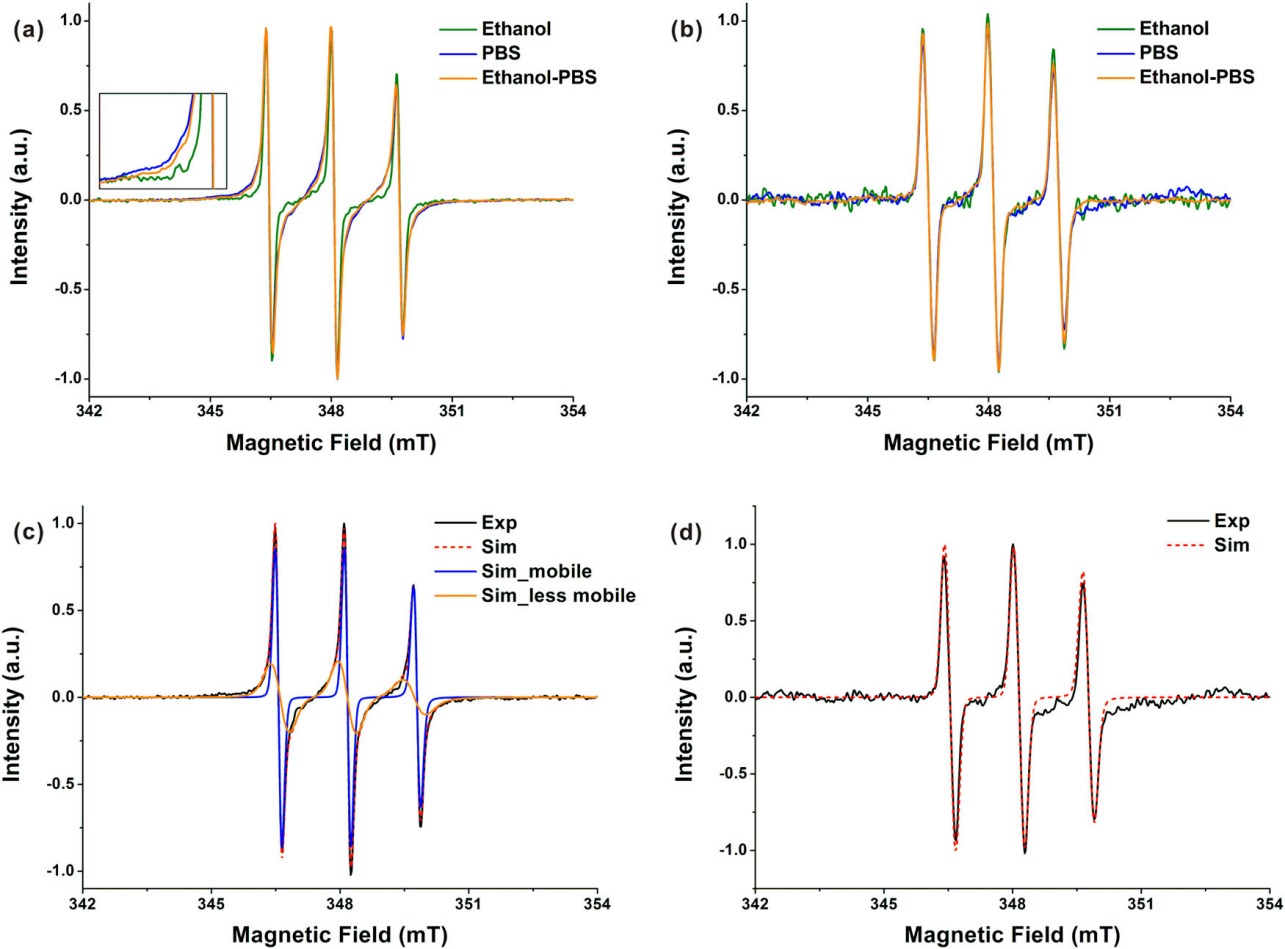
The averaged EPR spectra (*n* = 8) of PCA in **a** the whole skin containing the SC plus viable skin layers, and **b** only viable skin layers of excised human skin after the treatment with 0.4 % PCA solution dissolved in ethanol, PBS and ethanol-PBS (1:1, V/V), respectively. The inset visualizes the broadening of the spectra in Fig. 2a. The EPR spectra of PCA in porcine skin are presented in Fig. S1 of the Supplementary Material. Simulation examples of the EPR spectra of PCA in **c** the whole skin consisting of the SC plus viable skin layers, and **d** only viable skin layers of excised human skin exposed to 0.4 % PCA PBS solution, from which the fractions of PCA with high mobility in a hydrophilic microenvironment (PCA_mobile_) and PCA with lower mobility in a less hydrophilic microenvironment (PCA_less mobile_) were derived. The hyperfine coupling matrices (*a*_xx_, *a*_yy_, *a*_zz_) of (15 15 106) and (13 13 102) MHz, and the *g*-matrices (*g*_xx_, *g*_yy_, *g*_zz_) of (2.00805 2.00596 2.00212) and (2.00815 2.00596 2.00212) were used for the simulations of PCA_mobile_ and PCA_less mobile_, respectively. The rotational correlation time *T*_corr_ of PCA_mobile_ was about 0.1 ns and *T*_corr_ of PCA_less mobile_ was different for the cases of three solvents. The simulations of the EPR spectra of PCA in the skin treated with PCA dissolved in ethanol and ethanol-PBS (1:1, V/V), respectively, are illustrated in Fig. S2 of the Supplementary Material

Apart from the influences on the skin penetration amount and depth of PCA, solvent effects on the skin distribution of PCA were analyzed by interpreting the spectral shape with simulations of the EPR spectra. The magnetic and dynamic parameters obtained from the simulations could reveal the microenvironments around the PCA molecules in the skin, such as polarity and viscosity [20]. For both human and porcine skin, PCA in the whole skin (containing the SC and viable skin layers) exhibited different spectral broadening in the EPR spectra when using the three solvents (Fig. 2a and Fig. S1a of the Supplementary Material). The broadening decreased with the increase of ethanol concentration in the solvent. In contrast, PCA in the viable skin layers of both human and porcine skin showed similar EPR spectra among the cases of three solvents (Fig. 2b and Fig. S1b of the Supplementary Material). The results indicate that PCA had similar microenvironments in the viable skin layers regardless of the solvents because the SC barrier strongly prevented the solvents from entering the viable skin layers to alter the microenvironments of PCA there.

We assume that the spectral broadening could be due to the partitioning of PCA in two different skin microenvironments. Therefore, the spectra were simulated considering two components to get magnetic and dynamic parameters, i.e., the hyperfine coupling matrix (*a*_xx_, *a*_yy_, *a*_zz_), the *g*-matrix (*g*_xx_, *g*_yy_, *g*_zz_), and the rotational correlation time (*T*_corr_), among which *a*_zz_ and *g*_xx_ are sensitive to the changes of the microenvironmental polarity and *T*_corr_ reveals the mobility of the spin probe [39]. A higher *A*_zz_ together with a lower *g*_xx_ indicates a hydrophilic microenvironment, whereas vice versa a less hydrophilic or lipophilic microenvironment is present. A decrease of *T*_corr_ suggests higher mobility of the spin probe.

The simulation of the EPR spectrum of PCA in the whole human skin treated with PCA PBS solution was shown as an example owing to the remarkable spectral broadening (Fig. 2c). The simulation revealed that the spectrum comprised two kinds of spectra. As shown in Table 2 and Fig. 2c, the narrow spectrum represents PCA with the *T*_corr_ of about 0.1 ns, the higher hyperfine coupling constant of (15 15 106) MHz, and the lower *g*-matrix of (2.00805 2.00596 2.00212), which is attributed to PCA with high mobility in a hydrophilic microenvironment (PCA_mobile_). The broad spectrum represents PCA with the *T*_corr_ of about 0.7 ns, the lower hyperfine coupling constant of (13 13 102) MHz, and the higher *g*-matrix of (2.00815 2.00596 2.00212), which belongs to PCA with less mobility in a less hydrophilic microenvironment (PCA_less mobile_). The estimated *T*_corr_ of PCA_mobile_ was close to the *T*_corr_ of PCA in water (0.08 ns) [39]. Therefore, PCA_mobile_ could be localized in the water domains of corneocytes, intercellular regions, cytoplasm, etc [41].

Besides, the EPR spectrum of PCA in the viable skin layers was simulated (Fig. 2d), and PCA molecules were found to have the same *T*_corr_ and magnetic parameters as PCA_mobile_. It means that PCA in the viable skin layer was of high mobility and in a hydrophilic microenvironment (Table 2). PCA is generally considered as a cell membrane-impermeable probe due to its hydrophilicity [40]. However, several studies showed that PCA could enter cells, even though the intracellular amount was much lower than the intercellular one [48, 54]. Therefore, PCA in the hydrophilic microenvironment of the viable skin could be mostly distributed in the aqueous regions of the intercellular space and a few might be in the cytoplasm of the viable skin cells. As the above results have shown, PCA in the whole skin (containing the SC plus viable skin layers) comprised PCA_mobile_ and PCA_less mobile_, and PCA in the viable skin layers only included PCA_mobile_. Thus, PCA_less mobile_ can be assigned as PCA in the SC.

In addition, one of eight human skin samples and one of six porcine skin samples exposed to the PCA PBS solution could be simulated with three components, too (see Fig. S3 of the Supplementary Material). Besides PCA_mobile_ and PCA_less mobile_, the third component had the same magnetic parameters as PCA_less mobile_, while its *T*_corr_ was ten times slower than that of PCA_less mobile_ (6.3 ns), indicating reduced mobility of PCA (PCA_immobile_). Yet, the fraction of PCA_mobile_ did not change whether the EPR spectrum was simulated with two or three components (see Fig. S3 in the Supplementary Materials). Considering the same magnetic parameters and similar reduced mobility, the fractions of PCA_less mobile_ and PCA_immobile_ of the EPR spectra were summed up as the fraction of PCA_less mobile_ in the following calculation. The occurrence of PCA_immobile_ might be explained by the dryness of the skin sample. The SC hydration could be the most likely mechanism for PBS to deliver PCA into the skin [55]. In the time of sample processing after the incubation, PBS absorbed in the outmost layer of the SC might evaporate, leading to the immobilization of PCA in the upper SC layers.

#### Quantification of PCA in different skin microenvironments

**Fig. 3:**
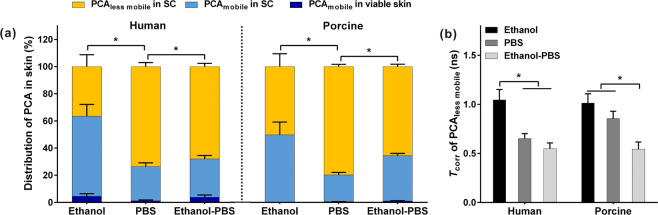
**a** Distribution of PCA_less mobile_ and PCA_mobile_ in the SC and viable skin after the application of 0.4% PCA solution dissolved in ethanol, PBS and ethanol-PBS (1:1, V/V), respectively, to human (*n* = 8) and porcine ear skin (*n* = 6), and **b** the rotational correlation time (*T*_corr_) of PCA_less mobile_ in the SC. Mean ± SEM, * indicates *p* < 0.05. No significant difference was found between human and porcine skin treated with the same PCA solution

The simulations of the EPR spectra of PCA in the whole skin provided the total fractions of PCA_mobile_ and PCA_less mobile_, respectively. The fraction of PCA_mobile_ in the viable skin layers was calculated by the amount of PCA in the viable skin layers and total PCA amount in the skin. Hence, the fraction of PCA_mobile_ in the SC is the difference between the total fraction of PCA_mobile_ and the fraction of PCA_mobile_ in the viable skin layers. In Fig. 3a, the PCA composition in the whole skin is shown. It consisted of PCA_mobile_ and PCA_less mobile_ for both human and porcine skin samples after exposure to the three PCA solutions, respectively. Concerning the skin distribution of PCA_mobile_, the majority of PCA_mobile_ was distributed in the SC, while only a few of PCA_mobile_ was in the viable layers skin. All PCA_less mobile_ were localized in the SC and the fraction of PCA_less mobile_ was increased with the decreased ethanol concentration in the solvent. From the solvent of ethanol to PBS, the fraction of PCA_less mobile_ in the human SC increased from 37 to 74%.

The *T*_corr_ of PCA_less mobile_ that indicates the mobility of PCA_less mobile_ in the SC is shown in Fig. 3b. For both human and porcine skin, the *T*_corr_ of PCA_less mobile_ when using ethanol as the solvent was significantly higher than the value in the case of ethanol-PBS (1:1), meaning that PCA_less mobile_ in the ethanol-treated SC had slower mobility. The evaporation of pure ethanol could cause dehydration of the SC, which could reduce the mobility of PCA_less mobile_ in the surrounding microenvironments. For ethanol-PBS (1:1), the addition of PBS not only reduced the evaporation of ethanol but also hydrated the SC, through which the skin penetration of ethanol could be enhanced [56]. Therefore, the lower *T*_corr_ of PCA_less mobile_ in the SC that treated with ethanol-PBS (1:1) could be due to the increased fluidity of the microenvironments around PCA_less mobile_ by the penetrated ethanol (for the localization of PCA_less mobile_ in the skin lipids, see the section of *Distributions of PCA in the SC*), leading to an increase in the mobility of PCA_less mobile_ [44, 57].

#### Distributions of PCA in the SC

**Fig. 4:**
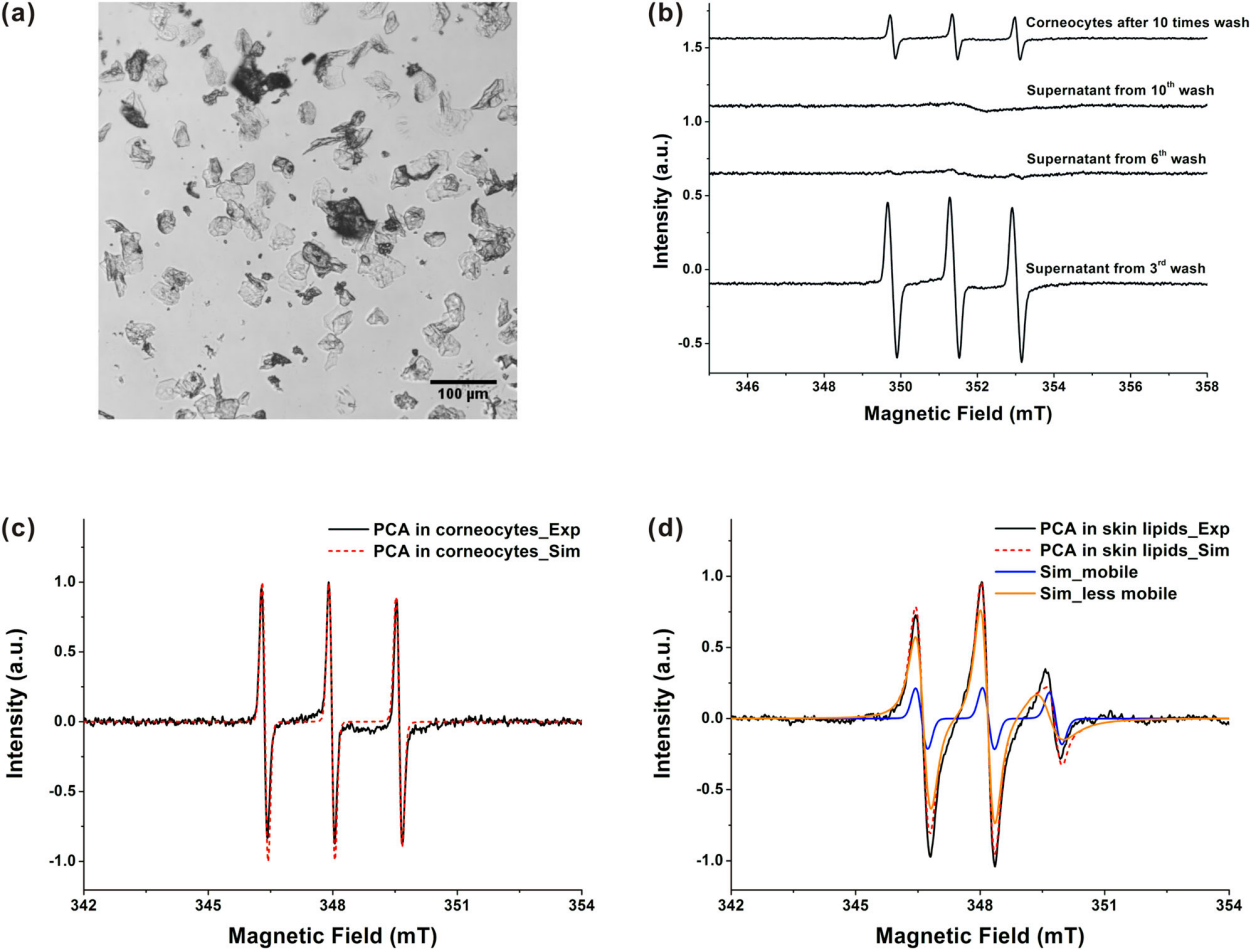
**a** The microscopic image of the morphology of corneocytes from porcine skin. Scale bar = 100 µm. **b** The EPR spectra of the supernatant of PBS used to wash corneocytes, and the precipitated corneocytes, which were incubated with 0.4% PCA ethanol-PBS (1:1, V/V) for 40 min at 32 °C. The experimental and simulated EPR spectra of PCA in (**c**) corneocytes and (**d**) skin lipids at the PCA concentration of 0.001% W/W

As the above results shown, PCA in the SC took up more than 95% of the total amount of PCA in the whole skin (Fig. 1), including all the PCA_less mobile_ and most PCA_mobile_ (Figs. 2c, d and 3a). Corneocytes and intercellular skin lipids mainly constitute the SC. Thus, the distributions of PCA_less- mobile_ and PCA_mobile_ in corneocytes and skin lipids were further investigated. First, corneocytes from the porcine SC were obtained using the detergent scrub method [34, 35]. As illustrated in Fig. 4a, most corneocytes were nearly elliptical with the conjugate diameters of 35 µm and 28 µm, which is in agreement with the published corneocyte diameter of 32 µm [58].

After incubation with 0.4% PCA ethanol-PBS (1:1), the corneocytes were washed with PBS ten times using centrifugation to remove the external PCA. In Fig. 4b, the EPR signal of PCA in the supernatant decreased with the number of washing cycles, indicating the removal of the external PCA. The supernatant from the 10th washing exhibited only a noise signal in its EPR spectrum, whereas the precipitated corneocytes after 10 times of washing showed a strong EPR signal of PCA. This indicates that PCA could diffuse into corneocytes. In Fig. 4c and Table 2, the simulation shows that PCA in corneocytes had the same hyperfine coupling constant and *g*-matrix as PCA_mobile_ in the skin. This reveals that PCA_mobile_ could be in the hydrophilic microenvironment of corneocytes. The only difference was the *T*_corr_ of PCA in corneocytes (0.03 ns), which was smaller than that of PCA_mobile_ in the skin, meaning that the mobility of PCA in corneocytes was faster (Table 1). The reason could be that the separated cornoecytes used in this experiment were more hydrated than those in the intact SC [41]. In addition, the detergent of Triton X-100 that was used to separate corneocytes might cause the structural changes of corneocytes, leading to an enhanced water diffusion into corneocytes.

The EPR spectrum of PCA in the extracted skin lipids was also investigated to mimic the microenvironments of intercellular skin lipids of the SC [33]. In Fig. 4d, the simulation shows that PCA in the skin lipids could have two kinds of microenvironments. About 91% of PCA in skin lipids had the same hyperfine coupling constant and *g*-matrix as PCA_less mobile_ in the SC (Table 2), indicating a less hydrophilic microenvironment. The *T*_corr_ of this part of PCA in the skin lipids (1.6 ns) was slightly larger than that of PCA_less mobile_ in the SC (Table 1). This could be due to that the lamellar structure of the intercellular skin lipids in the SC could be destroyed for the extracted skin lipids and different lipid packing orders might result in different *T*_corr_ values. The rest part of PCA in skin lipids (9%) were mobile in a hydrophilic microenvironment owing to the same magnetic parameters and *T*_corr_ as PCA_mobile_ in the skin. The hydrophilic microenvironments of the extracted skin lipids could be due to the water absorbed by the polar skin lipids, such as ceramide 1–3 and cholesteryl sulfate [59].

With the investigations of PCA in corneocytes and the skin lipids, PCA_less mobile_ in the SC could be attributed to PCA distributed in the intercellular skin lipids, and PCA_mobile_ in the SC could be predominately localized in corneocytes, as well as a few of them was in the intercellular skin lipids. Corresponding to the results in Fig. 3a, it could be deduced that with the increase of ethanol concentration in the solvent, the fraction of PCA in the less hydrophilic microenvironment of skin lipids (i.e., PCA_less mobile_) decreased, while the fraction of PCA in the hydrophilic microenvironments of corneocytes and skin lipids (i.e., PCA_mobile_) increased. Ethanol was found to perturb both the keratin structure of corneocytes and skin lipids, which might be a reason why ethanol facilitated the distribution of PCA in the hydrophilic microenvironments of corneocytes and skin lipids [45, 60].

## Conclusions

This work enhances the understanding of solvent effects on the skin penetration and spatial distribution of PCA. Poor skin penetration of PCA was observed when only ethanol was used, while it was increased 4.5-fold for PBS or ethanol-PBS (1:1). Among the three solvents, ethanol-PBS (1:1) delivered the most PCA into the viable skin layers, which could be sufficient to detect most part of free radicals produced in the viable skin layers. Nevertheless, more than 95% of the total PCA amount in the whole skin was accumulated in the SC, among which PCA with high mobility was predominantly distributed in the hydrophilic microenvironment of corneocytes and PCA with lower mobility was mainly distributed in the less hydrophilic microenvironment of intercellular skin lipids. A higher ethanol concentration in the solvent could improve the distribution of PCA in the hydrophilic microenvironments of the SC. The study not only suggests that ethanol-PBS (1:1) could be a suitable solvent to transport PCA into the skin but also provides valuable information for using PCA to study skin radical production (Table 2).

**Table 2:** The EPR magnetic and dynamic parameters of PCA in different biological media obtained from the simulations^a^

PCA in different biological media	PCA_mobile_			PCA_less mobile_		
	Hyperfine coupling constant (a*xx*, a*yy*, a*zz*) MHz	g-matrix (g*xx*, g*yy*, g*zz*)	Rotational correlation time T_corr_ (ns)	Hyperfine coupling constant (a*xx*, a*yy*, a*zz*) MHz	g-matrix (g*xx*, g*yy*, g*zz*)	Rotational correlation time T_corr_ (ns)
PCA in whole skin^b^	15	2.00805	0.1	13	2.00815	1.0–0.5
	15	2.00596		13	2.00596	
	106	2.00212		102	2.00212	
PCA in viable skin^b^	15	2.00805	0.1	No		
	15	2.00596				
	106	2.00212				
PCA in corneocytes	15	2.00805	0.03	No		
	15	2.00596				
	106	2.00212				
PCA in skin lipids^c^	15	2.00805	0.1	13	2.00815	1.6
	15	2.00596		13	2.00596	
	106	2.00212		102	2.00212	

^a^These are only estimated results from the simulations and used for the relative comparison

^b^Skin samples were treated with 0.4% PCA solution dissolved in ethanol, PBS, and ethanol-PBS (1:1, V/V), respectively

^c^The concentration of PCA in skin lipids was 0.001% W/W

## Supplementary Information

Suplementary Information
