# Correction to: Solvent Effects on Skin Penetration and Spatial Distribution of the Hydrophilic Nitroxide Spin Probe PCA Investigated by EPR

**DOI:** 10.1007/s12013-021-01041-5

**Published:** 2021-10-30

**Authors:** Pin Dong, Christian Teutloff, Jürgen Lademann, Alexa Patzelt, Monika Schäfer-Korting, Martina C. Meinke

**Affiliations:** 1 Department of Dermatology, Venereology and Allergology, Charité - Universitätsmedizin Berlin, corporate member of Freie Universität Berlin, Humboldt-Universität zu Berlin, and Berlin Institute of Health, Berlin, Germany; 2 Freie Universität Berlin, Institute of Pharmacy, Pharmacology and Toxicology, Berlin, Germany; 3 Freie Universität Berlin, Department of Physics, Institute of Experimental Physics, Berlin, Germany

Correction to: Cell Biochemistry and Biophysics (2020) 78:127–137

10.1007/s12013-020-00908-3

The article “Solvent Effects on Skin Penetration and Spatial Distribution of the Hydrophilic Nitroxide Spin Probe PCA Investigated by EPR” written by Pin Dong, Christian Teutloff, Jürgen Lademann, Dr. Alexa Patzelt, Monika Schäfer-Korting and Martina C. Meinke was originally published electronically on the publisher’s internet portal on 17 April 2021 without open access. With the author(s)’ decision to opt for Open Choice the copyright of the article changed on 15 October 2021 to © The Author(s) 2021 and the article is forthwith distributed under a Creative Commons Attribution 4.0 International License, which permits use, sharing, adaptation, distribution and reproduction in any medium or format, as long as you give appropriate credit to the original author(s) and the source, provide a link to the Creative Commons licence, and indicate if changes were made. The images or other third-party material in this article are included in the article’s Creative Commons licence, unless indicated otherwise in a credit line to the material. If material is not included in the article’s Creative Commons licence and your intended use is not permitted by statutory regulation or exceeds the permitted use, you will need to obtain permission directly from the copyright holder. To view a copy of this licence, visit http://creativecommons.org/licenses/by/4.0.

The original article has been corrected.

