# Supplementary material for: Solvent Effects on Skin Penetration and Spatial Distribution of the Hydrophilic Nitroxide Spin Probe PCA Investigated by EPR
Source: Cell Biochem Biophys. 2020 Apr 17;78(2):127–37. doi: 10.1007/s12013-020-00908-3 (PMC8557165; doi:10.1007/s12013-020-00908-3)
Supplement: Supplementary file 1 — Suplementary Information [file 12013_2020_908_MOESM1_ESM.docx]

**Supplementary material**

**Solvent effects on skin penetration and distribution of the hydrophilic nitroxide spin probe PCA investigated by EPR**

Pin Dong ^ab^, Christian Teutloff ^c^, Jürgen Lademann ^a^, Alexa Patzelt ^a^, Monika Schäfer-Korting ^b^, Martina C. Meinke ^a^.

^a^ Charité - Universitätsmedizin Berlin, corporate member of Freie Universität Berlin, Humboldt-Universität zu Berlin, and Berlin Institute of Health, Department of Dermatology, Venereology and Allergology, Berlin, Germany

^b^ Freie Universität Berlin, Institute of Pharmacy, Pharmacology and Toxicology, Berlin, Germany

^c^ Freie Universität Berlin, Institute of Experimental Physics, Department of Physics, Berlin, Germany


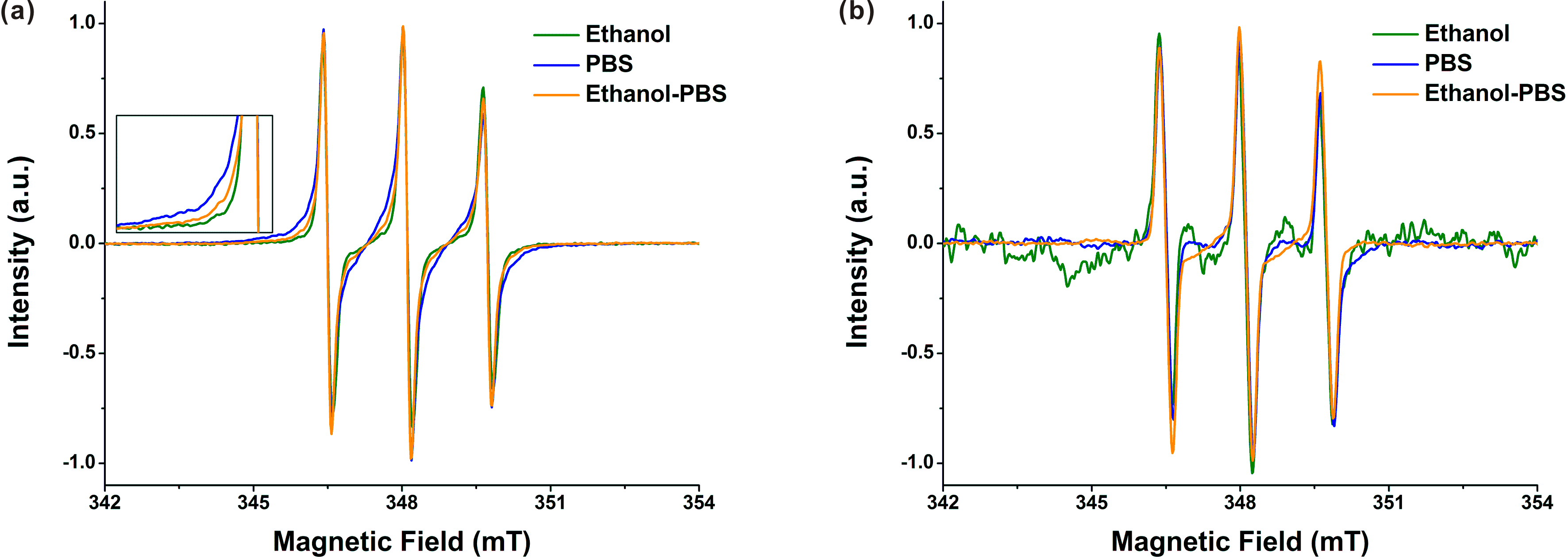


**Fig. S1** The averaged EPR spectra (*n* = 6) of PCA in the (a) whole skin containing the SC plus viable skin and (b) viable skin of porcine ear skin after treated with 0.4 % PCA dissolved in ethanol, PBS and ethanol-PBS (1:1, V/V), respectively. The inset is a magnified spectral part.


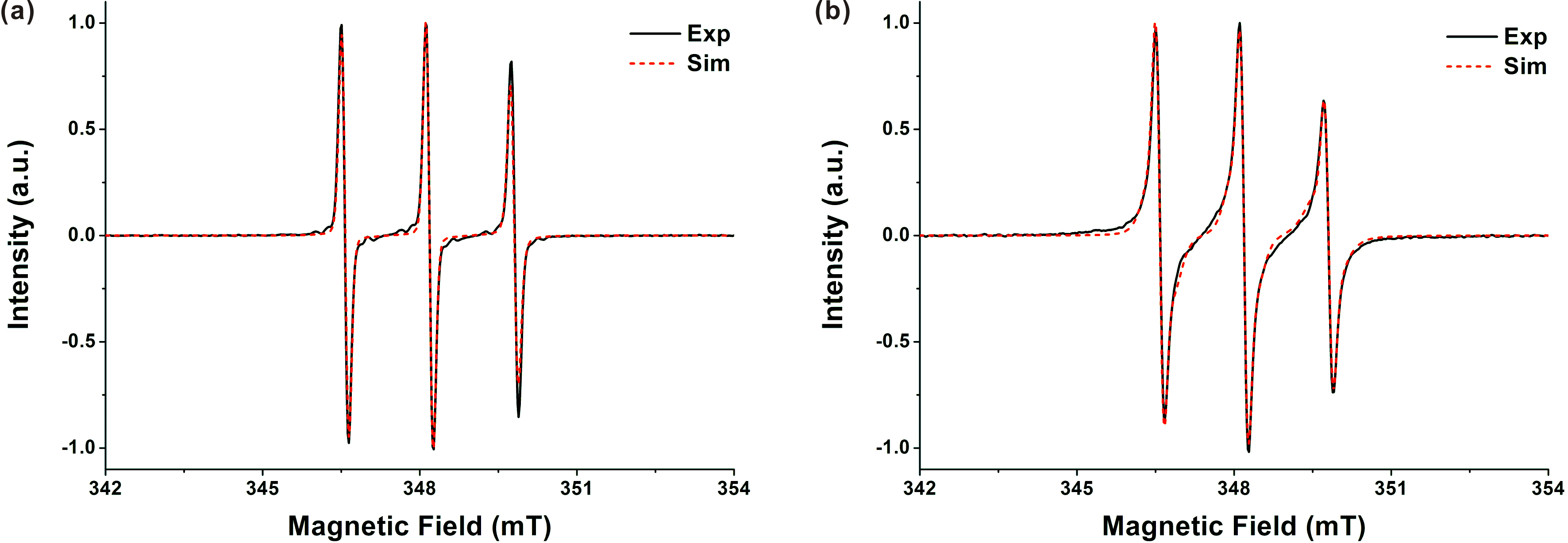


**Fig. S2** Simulation examples of the EPR spectra of PCA in the whole skin containing the SC plus viable skin of human skin after applied 0.4 % PCA dissolved in (a) ethanol and (b) ethanol-PBS (1:1, V/V) respectively, from which the fractions of PCA with high mobility in a hydrophilic microenvironment (PCA_mobile_) and PCA with less mobility in a less hydrophilic microenvironment (PCA_less mobile_) were derived. The hyperfine coupling matrices (*a*_xx_, *a*_yy_, *a*_zz_) of (15 15 106) and (13 13 102) MHz, and the *g*-matrices (*g*_xx_, *g*_yy_, *g*_zz_) of (2.00805 2.00596 2.00212) and (2.00815 2.00596 2.00212) were used for the simulations of PCA_mobile_ and PCA_less mobile_, respectively. The rotational correlation time of PCA_mobile_ was about 0.1 ns.


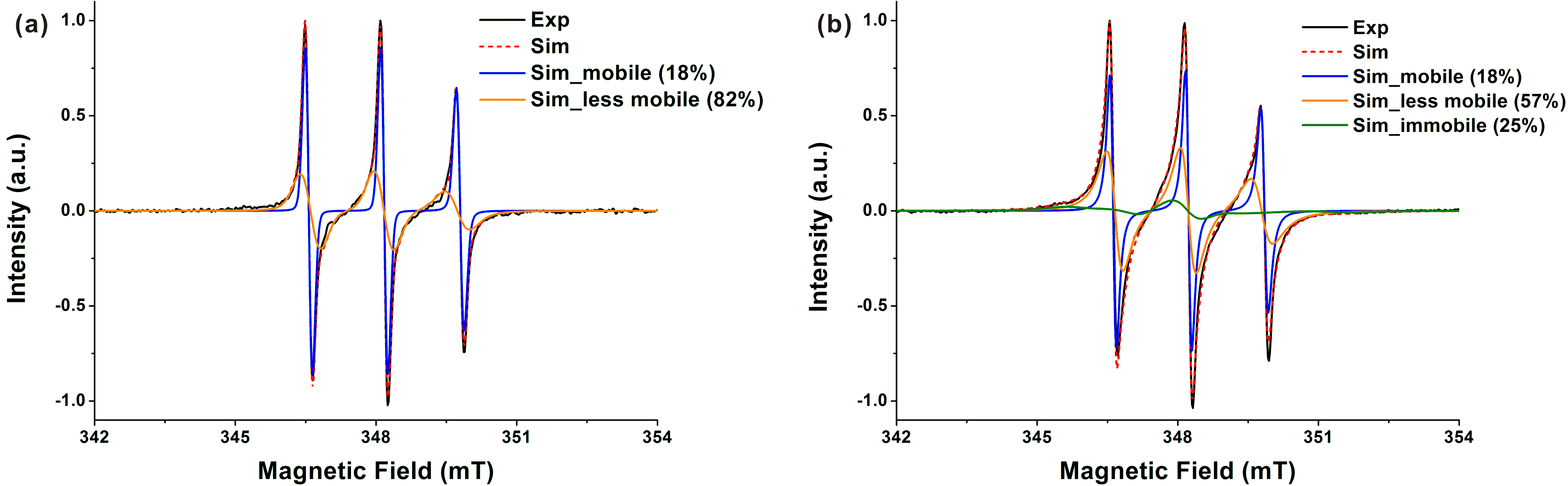


**Fig. S3** The EPR spectrum of PCA in one human skin sample containing the SC and viable skin exposed to 0.4% PCA PBS was simulated as (a) two components and (b) three components. The values in the brackets represent the fractions of the components. When simulating as two components, the hyperfine coupling matrices (*a*_xx_, *a*_yy_, *a*_zz_) of (15 15 106) and (13 13 102) MHz, the *g*- matrices (*g*_xx_, *g*_yy_, *g*_zz_) of (2.00805 2.00596 2.00212) and (2.00815 2.00596 2.00212), the correlation time of 0.1 ns and 0.7 ns were used for the simulations of PCA_mobile_ and PCA_less mobile_, respectively. When including the third component, its magnetic parameters were the same as those of PCA_less mobile_, and only its rotational correlation time is 6.3 ns. Thus, the third component can be regarded as PCA with slow mobility in the less hydrophilic microenvironment (PCA_immobile_). Here, the summed up fraction of PCA_less mobile_ and PCA_immobile_ was equal to the fraction of PCA_less mobile_ when simulating the spectrum as two components. Therefore, the fraction of PCA_mobile_ did not change in both simulation strategies.
